# Biochar-Mediated Degradation of Roxarsone by *Shewanella oneidensis* MR-1

**DOI:** 10.3389/fmicb.2022.846228

**Published:** 2022-03-14

**Authors:** Li Wengang, Chen Fang, Zhong Rong, Chen Cuihong

**Affiliations:** ^1^Key Laboratory of Pollution Processes and Environmental Criteria (Ministry of Education)/Tianjin Engineering Center of Environmental Diagnosis and Contamination Remediation, College of Environmental Science and Engineering, Nankai University, Tianjin, China; ^2^School of Resources and Materials, Northeastern University at Qinhuangdao, Qinhuangdao, China; ^3^Tangshan Ecological Environmental Bureau, Tangshan, China

**Keywords:** kinetics, biochar, *Shewanella oneidensis* MR-1, roxarsone, transformation

## Abstract

It is widely believed that biochar plays an essential role in sequestrating pollutants. The impacts of biochar on microbial growth, and consequently on the environmental fate of pollutants, however, remains poorly understood. In this study, wheat-straw-derived biochar was used to investigate how biochar amendment affected *Shewanella oneidensis* MR-1 growth and roxarsone transformation in water under anaerobic conditions. Three biochar with different physicochemical properties were used to mediate the roxarsone degradation. The results showed that the degradation rate of roxarsone could be accelerated by the increase of biochar pyrolysis temperature. From the characterization of biochar, the total specific surface area, micropore surface area and micropore volume of biochar increase, but the average pore diameter decreases as the pyrolysis temperature increases. Through infrared spectroscopy analysis, it was found that as the pyrolysis temperature increases, the degree of condensation of biochar increases, thereby increasing the pollutant removal rate. From the changes of the relative concentration of MR-1 and its secreted extracellular polymer content, the growth promotion ability of biochar also increases as the pyrolysis temperature increases. These results suggest that wheat-straw-derived biochar may be an important agent for activating microbial growth and can be used to accelerate the transformation of roxarsone, which could be a novel strategy for roxarsone remediation.

## Introduction

As human demand for meat continues to increase, the poultry industry has gradually become one of the fastest-growing agricultural sectors in decades. Through the extensive application of veterinary drugs and feed additives, production efficiency and product quality have been improved (Sarmah et al., 2006). Roxarsone (3-nitro-4-hydroxyphenylarsine) was first produced 70 years ago, which was used as feed additives to control coccidial intestinal parasites and prevent parasitic infections and improve the pigmentation of meat (Silbergeld and Nachman, 2008; Nachman et al., 2013). The use of roxarsone in the poultry industry has been banned in most developed countries, including China, but long-term extensive usage has resulted in a massive accumulation of roxarsone in the environment (Huang et al., 2019; Wang G. et al., 2020). Roxarsone is barely decomposed within the animal body, and is usually excreted as its initial form along with the animal waste (Makris et al., 2008). Along with the storage or field application of animal waste or direct discharge into the environment, roxarsone can easily enter into the surface- or ground-water and soil due to its water-soluble capacity (Silbergeld and Nachman, 2008; Huang et al., 2019). Roxarsone can be oxidized, reduced, methylated or demethylated through numerous physical, chemical and biological interactions, and eventually produce a variety of arsenic compounds (Garbarino et al., 2003; Chen et al., 2016; Han et al., 2017; Oyewumi and Schreiber, 2017).

*Shewanella* species are widely distributed in freshwater, marine, soil and sedimentary, environments (Heidelberg et al., 2002; Harris et al., 2010). Many strains of *Shewanella* species can couple anaerobic growth with the respiratory reduction of minerals and other high-redox potential contaminants (Aulenta et al., 2013; Shi et al., 2016; Wang H. F. et al., 2020). To accomplish this coupling, *Shewanella* strains usually metabolize substrates to generate electrons and transport them to the outer surface of the membrane, where contaminants are reduced directly or indirectly (Kato et al., 2012; Shi et al., 2016; Wang H. F. et al., 2020). Some organic matter, such as biochar and humic substances, can mediate electron transfer at the interface between microbes and acceptors, thus accelerating the biochemical transformation (Klüpfel et al., 2014b; Pignatello et al., 2017; Stern et al., 2018; Wang H. F. et al., 2020).

Natural organic matter (NOM)-mediated microbial redox reactions are important for the biogeochemical cycles of carbon and redox-active compounds. Biochar, a significant fraction of natural organic matter, is produced by incomplete combustion and usually has the advantages of eco-friendly properties, reusability and cost-effectiveness (Kappler et al., 2014; Hemavathy et al., 2020; Gayathri et al., 2021). Biochar usually have a highly porous structure, multiple surface functional groups, and condensed aromatic structures, these properties confer biochar with great potential for many environmental and ecological application (Ahmad et al., 2014; Xu et al., 2019; Wang H. F. et al., 2020). Biochar not only could accept or release electrons (Klüpfel et al., 2014a). It also could act as an electron mediator (i.e., electron shuttle) to accelerate redox reactions (Saquing et al., 2016). Previous studies have shown that biochar can facilitate the biochemical degradation of toxic organic compounds (Zhang et al., 2019; Wang H. F. et al., 2020). The surface functional groups and condensed aromatic structures constitute the electroactive components in biochar (Sun et al., 2017). However, limited knowledge is available for the role of biochar in the redox reaction for roxarsone.

Therefore, the central goal of this work is to compare roxarsone removal by *Shewanella oneidensis* MR-1 in the presence and absence of biochar, and to elucidate the influence of biochar on the biotransformation of roxarsone.

## Materials and Methods

### Materials

The raw material for preparing biochar in this experiment was wheat straw (from Hebei Province, China). The preparation method of biochar adopts the method in references (Zuriaga-Agustí et al., 2013; Lyu et al., 2016). Briefly, the dried wheat straw powder was heated at 300, 500, and 600°C for 2 h in a stainless-steel reactor in a Muffle Furnace under the O_2_-limited condition. After it was cooled to room temperature, and then passed through a 100-mesh sieve and washed with deionized water until the biochar was neutral. Keep it in a sealed bag and place it in a desiccator for later use. Biochar prepared at 300, 500, and 600°C were termed 300BC, 500BC and 600BC.

*Shewanella oneidensis* MR-1 was cultivated in LB medium at 30°C. After incubation for 16 h, 50 mL of the culture was centrifuged (6,000 *g* for 5 min), and the cell pellet was washed twice with anoxic bicarbonate buffer (*pH* 7) and suspended in the same buffer.

### Methods

Microbial degradation experiments were conducted in 50 mL anaerobic bottles. The experiments were designed into 5 groups, divided into biochar combined with MR-1 group (called 300BC biotic, 500BC biotic, and 600BC biotic), MR-1 alone group (called only MR-1), and blank group. Each group has three parallel samples. The initial concentration of roxarsone was 1.0 mmol/L, the initial concentration of MR-1 (OD600) was 0.8, and 5 mmol/L of sodium lactate was added as an exogenous carbon source. The N_2_/CO_2_ (80:20) mixed gas was purged into the butyl-stopper glass bottles for 15 min to remove oxygen. The anaerobic flask was placed in a constant temperature shaker under dark conditions. The parameters of the shaker were set to 100 rpm and 30°C. When the reaction time was 12, 24, 36, 48, 84, 132, 168, and 192 h, the concentration of roxarsone in the sample was analyzed. When the reaction time was 0, 48, 72, and 100 h, the extracellular polymer was extracted, and the protein and polysaccharide content were determined.

In this experiment, the concentration of roxarsone was determined by a high-performance liquid chromatograph (Flexar, PerkinElmer, United States), and the wavelength was 264 nm. The chromatographic column used in the determination was a C18 column (4.6 mm × 150 mm). The mobile phase was composed of 0.05 mol/L potassium dihydrogen phosphate (KH_2_PO_4_), methanol, and 10% glacial acetic acid (V/V). The volume ratio was 95:5:0.1, and the mobile phase flow rate was set to 1.0 mL/min. The column temperature was 30°C.

Extracellular polymeric substances (EPS) was extracted by an improved cation exchange resin method (Liang Z. W. et al., 2010; Zuriaga-Agustí et al., 2013). 10 mL of bacterial suspension was placed in a centrifuge tube, centrifuged at 2,000 *g* for 15 min at 4°C, and the supernatant was discarded. Resuspend the pellet with 10 mL PBS buffer. Add 1 g of sodium type 732 type cation exchange resin (20–50 mesh) to the suspension, which was shaken at 40 rpm for 6 h at 4°C, and then centrifuged for 15 min with a high-speed centrifuge. The centrifuge parameters were set to 4°C and 10,000 *g*. After centrifugation, the supernatant was filtered with a 0.22-μm polyethersulfone filter, and stored at −20°C for testing. The sulfuric acid-anthrone method was used to determine the polysaccharides in EPS (Yuan et al., 2011). The BCA protein concentration determination kit was used to detect the protein content. To determine the relative concentration of MR-1 in different groups, 5 mL samples were taken from the treatment groups at 24, 48, and 84 h, the OD600 value was measured with a microplate reader.

The physical and chemical properties of biochar samples (300BC, 500BC, and 600BC) were analyzed by JOEL JSM-7800F field emission scanning electron microscope (FE-SEM), American Mike ASAP 2460 multi-station extended automatic rapid surface area analyzer, the Nicolet iS50 Fourier transform infrared spectrometer. The surface group in biochar samples are analyzed by solid-state ^13^C nuclear magnetic resonance (NMR) spectroscopy on a 400 MHz NMR spectrometer (Advance III WB 400, Bruker, Germany).

The degradation rates of roxarsone by MR-1 in the presence and absence of biochar were analyzed by one-way analysis of variance (ANOVA) followed by Duncan test using SPSS 27.0 software. The results were considered significant when the *p* value was less than 0.05.

## Results and Discussion

### Roxarsone Transformation Kinetics in the Presence of Biochar and MR-1

**FIGURE 1:**
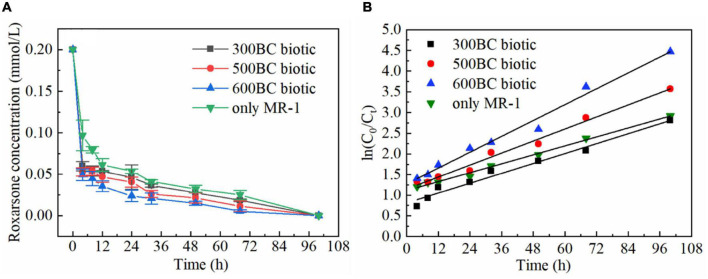
Biotransformation of roxarsone by MR-1 in the presence and absence of biochar. **(A)** The concentration of roxarsone within reaction time. **(B)** The change of ln(C_0_/C_*t*_) with reaction time.

The biotransformation of roxarsone in the presence and absence of biochar was shown in Figure 1. According to the results in Table 1, roxarsone was almost completely degraded in all groups within 68 h. It was found that the degradation rates of roxarsone at 68 h were: 600BC biotic > 500BC biotic > 300BC biotic > only MR-1, and the degradation rates were increased by 9.79, 6.84, and 3.23% with the addition of 600BC, 500BC and 300BC. Therefore, biochar had a promoting effect on removing roxarsone by MR-1. The ability of biochar to promote the degradation of roxarsone by MR-1 was followed as: 600BC > 500BC > 300BC. It was found that the change of ln(C_0_/C_*t*_) with time (t) showed a good linear relationship (C_0_ is the initial concentration of roxarsone, C_*t*_ is the concentration of roxarsone remaining in the reaction when the reaction time is t), in line with the first-order kinetic reaction fitting equation. The kinetic curves of the removal of roxarsone by MR-1 in the presence and absence of biochar were shown in Figure 1B. The average rate constant fitted by the first-order kinetic equation was 0.017 h^–1^ in the only MR-1 group, and the removal rates of roxarsone by MR-1 combined with biochar group were greater than that of only MR-1 group. According to the results in Table 2 the average rate constants in MR-1 combined with 300BC, 500BC, and 600BC groups were 0.020, 0.024, and 0.032 h^–1^, which showed the order: 600BC biotic > 500BC biotic > 300BC biotic. It can be seen that when the pyrolysis temperature is in the range of 300–600°C, the higher the pyrolysis temperature of biochar, the greater the promotion effect of biochar on the removal of roxarsone by MR-1. Lehmann et al. (2011) summarized a large amount of literature and found that the addition of biochar in most studies can increase the biomass of microorganisms. On the other hand, Klüpfel et al. (2014b) found that in the process of biochar combined with MR-1 for oxidation-reduction, the biochar produced at the medium to high temperature pyrolysis temperature (400∼600°C) showed the highest ability to accept and supply electrons, while the low-temperature pyrolysis temperature (300°C) biochar has the worst effect. Because the pyrolysis temperature of biochar was related to its transfer rate of electrons, it affected the speed of MR-1’s reaction to remove roxarsone. Zhang et al. (2019) found that with the increase of pyrolysis temperature, the conversion ability of biochar was gradually changing from the supply of electrons to the dominance of electrons. The surface functional groups and condensed aromatic structure play an important role in the reaction.

**TABLE 1:** Degradation rates of roxarsone by MR-1 in the presence and absence of biochar.

Time/h	300BC biotic	500BC biotic	600BC biotic	only MR-1
4 h	70.04% d	73.53% c	75.29% b	51.61% a
68 h	90.76% b	94.37% c	97.32% d	87.53% a

*Different small letters in the same time refer to the difference at significance level p < 0.05.*

**TABLE 2:** Kinetic parameters of roxarsone removal by MR-1 in the presence and absence of biochar.

	Rate constant K (h^–1^)	R^2^
Only MR-1	0.017 ± 0.0006	0.98
300BC biotic	0.020 ± 0.0011	0.99
500BC biotic	0.024 ± 0.0016	0.98
600BC biotic	0.032 ± 0.0012	0.99

### Extracellular Polymeric Substances Changes of MR-1 Under Different Biochar

**FIGURE 2:**
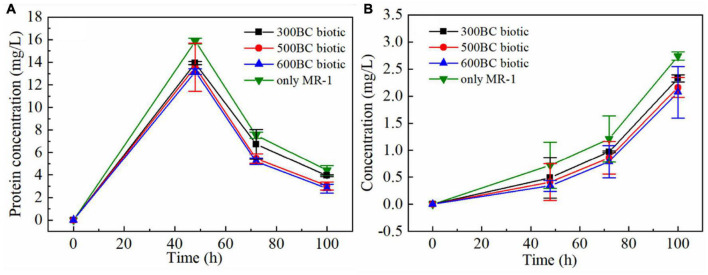
Changes of protein **(A)** and polysaccharides **(B)** of MR-1 in the presence and absence of biochar.

In order to explore the effect of biochar on the secretion of the extracellular polymer by MR-1, the content of protein and polysaccharides at 48, 72, and 100 h in different treatments were measured, as shown in the Figure 2. It can be seen that the changing trend of the protein concentration and polysaccharides concentration was the same in the presence and absence of biochar. At the same time, the polysaccharides and protein content in the only MR-1 group was higher than that of biochar combined with MR-1 group. And in the presence of biochar, the polysaccharide and protein content in the 300BC biotic group was the highest, and that in the 600BC biotic group was the lowest. Therefore, the higher the pyrolysis temperature of the biochar, the lower the content of polysaccharides and proteins produced by MR-1 during the reaction. Because the poorer the living environment of MR-1 was, the more extracellular polymers were secreted, MR-1 can adapt to the surrounding environment to ensure survival (Vu et al., 2009). The results showed that MR-1 survives well in the presence of biochar, and the growth environment of 600BC was the best, followed by the growth environment of 500BC, and the growth environment of 300BC was the worst. This was because, on the one hand, biochar can provide nutrients for microorganisms; on the other hand, because of its large surface area, porous structure, and strong affinity for microorganisms, biochar can be used as a habitat for microorganisms (Lehmann et al., 2011; Wahla et al., 2020).

### The Relative Concentration Changes of MR-1 Under Different Biochar

**FIGURE 3:**
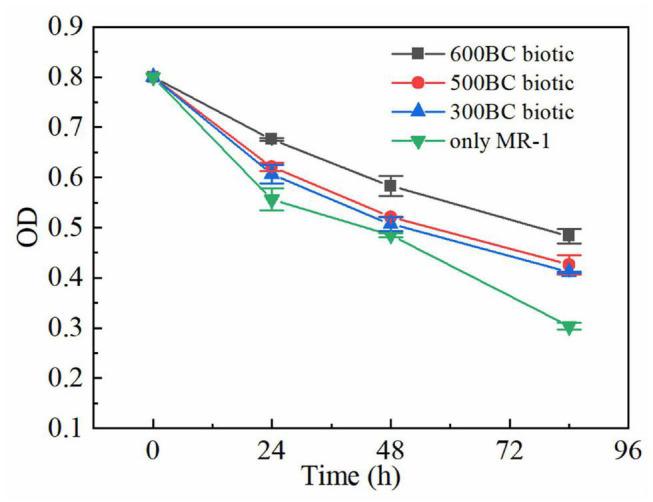
Changes of OD in the presence and absence of biochars.

As shown in Figure 3, the OD values in the presence and absence of biochar decreased as the reaction time lasted, which inferred that the MR-1 is in the stationary and decline phase and the proliferation slowed down as the degradation of roxarsone. The OD values in the only MR-1 group decreased fastest. In the presence of biochar, the OD value of MR-1 in the whole reaction process was sorted: 600BC biotic group > 500BC biotic group > 300BC biotic group. Moreover, when the reaction reached 84 h, the OD values in the presence of biochar were much greater than that of only MR-1 group, especially the OD value of the 600BC biotic group was the largest. The living or dead states of MR-1 in the presence and absence of biochar was determined by Live/Dead assay using a laser scanning confocal microscope when the reaction reached 55 h (Supplementary Figure S1). In 600BC biotic group, most of the MR-1 exhibit green fluorescence, which indicated that the number of live MR-1 was much larger than the dead MR-1, and the growth activity is the best compared to other groups. The addition of biochar made the bacteria grow better, and the activity was better. The addition of biochar benefited to the MR-1 growth, which was consistent with previous research results. Liang B. Q. et al. (2010) studied the effect of biochar on microorganisms in soil, and found that the biomass of microorganisms in soils with high biochar content was 125% higher than that in adjacent soils with low biochar content.

### The Physical and Chemical Properties of Biochar

#### Microscopic Morphology of Biochar

**FIGURE 4:**
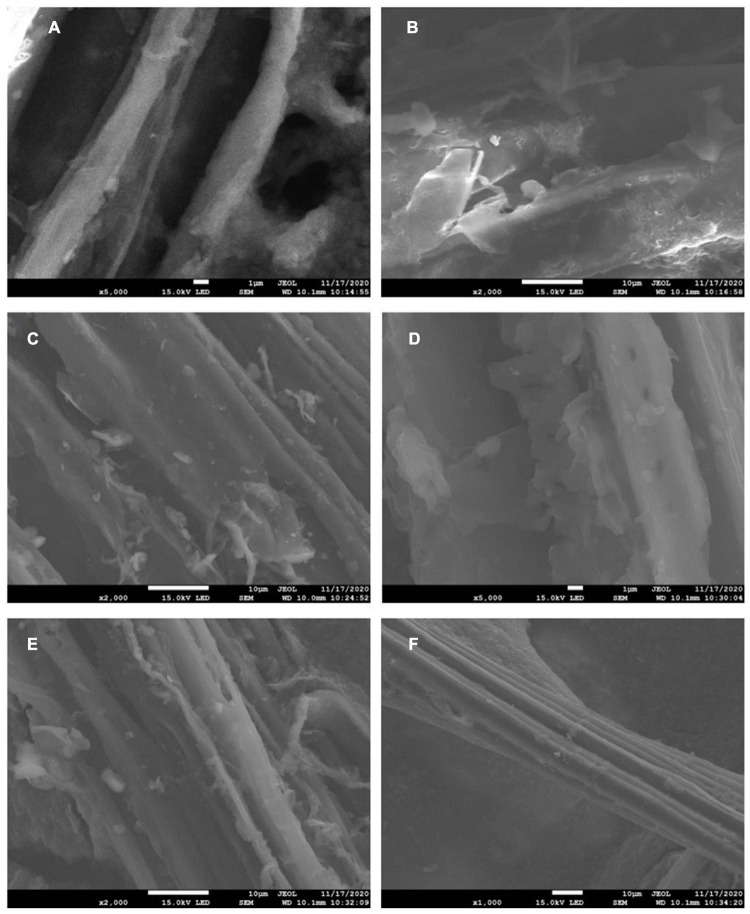
Electron micrographs of biochar at different pyrolysis temperatures before the reaction [panels **(A,B)** are 300BC; panels **(C,D)** are 500BC; panels **(E,F)** are 600BC].

Figure 4 was a scanning electron micrograph of biochar prepared under the conditions of pyrolysis temperature of 300, 500, and 600°C. It can be seen from the figure that there were wrinkles, micropores, different pore structures and morphologies on the surface of biochar. When the pyrolysis temperature was 300°C, there were fewer particles and massive debris on the surface of biochar. When the pyrolysis temperature rose to 500 and 600°C, cellulose, lignin, and other components in biomass were decomposed gradually with the increase of pyrolysis temperature, and the surface of biochar became more wrinkled at 600°C. Therefore, the pyrolysis temperature had a great influence on the surface microstructure of biochar.

#### Analysis of Specific Surface Area and Pore Structure

In order to further understand the difference in morphology of biochar at different pyrolysis temperatures, the specific surface area and pore structure of the biochar were analyzed by the BET specific surface area analyzer. The specific surface area, pore volume, and pore size of the biochar at different pyrolysis temperatures were measured, which was shown in Table 3. Compared with 300BC, when the pyrolysis temperature was increased to 600°C, the specific surface area of biochar increased significantly. The total specific surface area increased from 2.08 to 162.04 m^2^/g. The micropore surface area occupied 79.3% of the total specific surface area, indicating the existence of a large number of internal pores. And the average pore diameter decreased with the increase of pyrolysis temperature. When the pyrolysis temperature increased from 300 to 500°C, the pore volume of micropores increased significantly, from 0.0004 to 0.0132 cm^3^/g, an increase of more than 30 times. In the pyrolysis temperature range of 500–600°C, the specific surface area and pore volume of biochar increased, which may be due to the decomposition of cellulose and fat of wheat straw. Chang et al. (2017) found that the higher the temperature in the biochar preparation process, the larger the internal pore volume. when the pyrolysis temperature rose from 200 to 600°C, the number of micropores in the prepared biochar also increased significantly.

**TABLE 3:** Specific surface area, pore volume, and pore diameter of biochar.

Sample	Total specific surface area (m^2^/g)	Micropore surface area (m^2^/g)	Outer surface area (m^2^/g)	Micropore volume (cm^3^/g)	BET average pore diameter (nm)
BC300	2.08	1.32	0.76	0.0004	7.22
BC500	41.27	32.99	8.28	0.0132	3.17
BC600	162.04	128.53	33.51	0.0511	2.13

When biochar was applied to the soil, its pore size may have an extremely important effect on the growth of MR-1. Researchers have speculated that both bacteria and fungi can better defend against competitors by exploring the pore environment in biochar. Although there is currently no quantifiable evidence to prove that the pores of biochar have a protective effect on microorganisms, the pore size distribution of biochar and microorganisms, as well as the visually detectable results, provide a basis for this hypothesis. There has been some evidence that pore size has a significant influence on the retention of microorganisms (Saito and Marumoto, 2002; Lehmann et al., 2011).

#### Infrared Spectroscopy Analysis of Biochar

**FIGURE 5:**
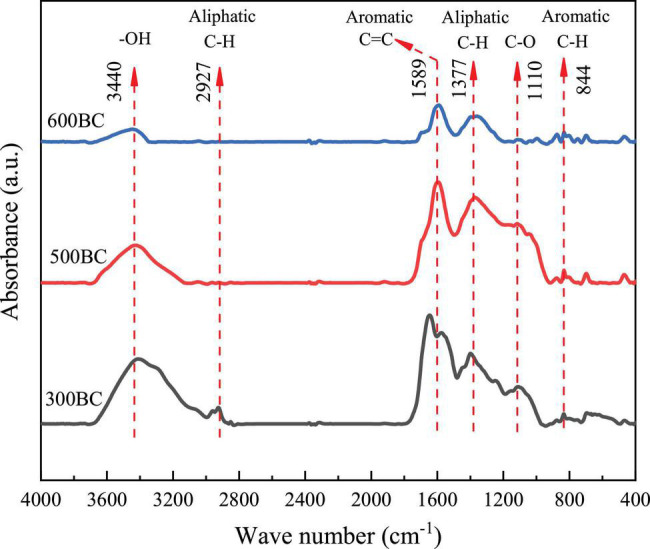
Infrared spectra of biochar prepared at different pyrolysis temperatures.

The Fourier infrared transform spectra of 300BC, 500BC, and 600BC were shown in Figure 5, respectively. First, the peak at 3,430 cm^–1^ was considered to be caused by the stretching vibration of OH. The 2,924–2,927 cm^–1^ and 1,377 cm^–1^ represented the aliphatic C-H stretching vibration absorption peaks on the biopolymer (Such as cellulose, hemicellulose, and lignin), 1589 cm^–1^ was aromatic C = C absorption peak, and 1,110 cm^–1^ was the CO stretching vibration absorption peak of hydrocarbons (Schwanninger et al., 2004; Tatzber et al., 2009). Finally, at 788–881 cm^–1^ was the plane vibration of C-H on aromatic carbon. With the increase of pyrolysis temperature, the aliphatic C and O-containing functional groups in the hemicellulose, cellulose, and lignin in the biomass gradually disappear. The absorption peak of -OH at 3,440 cm^–1^ gradually decreased, and that of C=C and C-H on aromatic carbon increased. Further, the NMR spectroscopy also reveal an enhancement of aromatic C=C and ketone groups (C=O) with increasing pyrolysis temperature (Supplementary Figure S2). Both aromatic C=C and C=O are necessary for high-performance electron exchange capacity (Wang H. F. et al., 2020). The temperature was positively related to the aromatic cluster size (Cao et al., 2012). Based on these, it can be inferred that as the degree of condensation of biochar increased, the ability of the electron shuttle may increase, thereby increasing the pollutant removal rate. However, the “mediator mechanism” of biochar need further study.

## Conclusion

In this paper, roxarsone biotransformation was promoted by the biochar. The higher the pyrolysis temperature of biochar, the higher the biotransformation rate. When the pyrolysis temperature increases, the pore volume and specific surface area of biochar increase, and MR-1 grows better. Through infrared spectroscopy and NMR analysis, it can be seen that as the pyrolysis temperature increases, the degree of condensation of biochar increases, thereby increasing the removal rate of pollutants. However, the mechanism of biochar mediating the biochemical transformation of roxarsone needs more attention. The transformation mechanism of roxarsone was not studied in this paper, which was important in the arsenic geochemical cycle.

## Data Availability Statement

The original contributions presented in the study are included in the article/Supplementary Material, further inquiries can be directed to the corresponding author.

## Author Contributions

L-WG and C-CH designed the research. L-WG and Z-R performed the research. L-WG, C-CH, and C-F analyzed the data, and wrote the manuscript with input from all authors.

## Conflict of Interest

The authors declare that the research was conducted in the absence of any commercial or financial relationships that could be construed as a potential conflict of interest.

## Publisher’s Note

All claims expressed in this article are solely those of the authors and do not necessarily represent those of their affiliated organizations, or those of the publisher, the editors and the reviewers. Any product that may be evaluated in this article, or claim that may be made by its manufacturer, is not guaranteed or endorsed by the publisher.
