# Supplementary material for: Biochar-Mediated Degradation of Roxarsone by Shewanella oneidensis MR-1
Source: Front Microbiol. 2022 Mar 14;13:846228. doi: 10.3389/fmicb.2022.846228 (PMC8964303; doi:10.3389/fmicb.2022.846228)
Supplement: Supplementary file 1 [file Data_Sheet_1.docx]

**Live/Dead assay by a laser scanning confocal microscope**

In order to explore the effect of biochar at different pyrolysis temperatures on the activity of MR-1, the live or dead states of MR-1 was observed when the reaction time reached 55 h. The sample processing method of the laser scanning confocal microscope was as follows: take 1 mL bacterial solution in the anaerobic bottle from the different treatments, and add 1 mL PBS to wash, then repeat the washing for 3 times. Mix 50 μL bacterial solution and 20 μL SYTO9/PI dye mixture, and store in tin foil for 20 min in the dark. And then take 10 μL on the glass slide, cover it with a glass cover slip. The fluorescence from live or dead cells was observed using a laser scanning confocal microscope (LSM880, Zeiss, Germany). The excitation wavelengths were 488 nm (names as “green fluorescence channel”) and 543 nm (named as “red fluorescence channel”). The living bacteria was evaluated by the signal from the green fluorescence channel and the dead states by the signal from the red fluorescence channel.


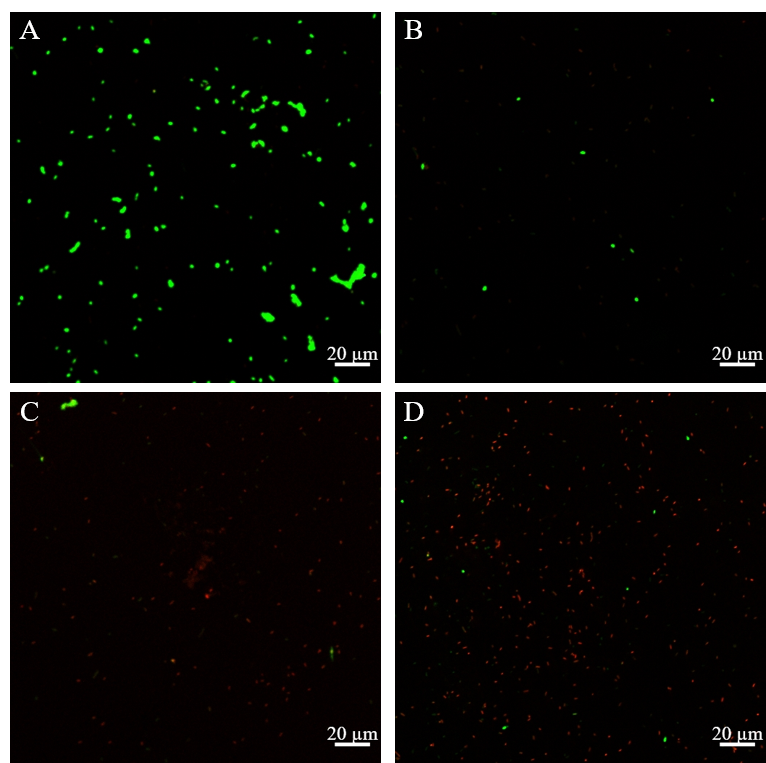


Figure S1. Living or dead state of MR-1 determined from Live/Dead assay with a laser scanning confocal microscope. (A) 600BC biotic, (B) 500BC biotic, (C) 300BC biotic, and (D) only MR-1.


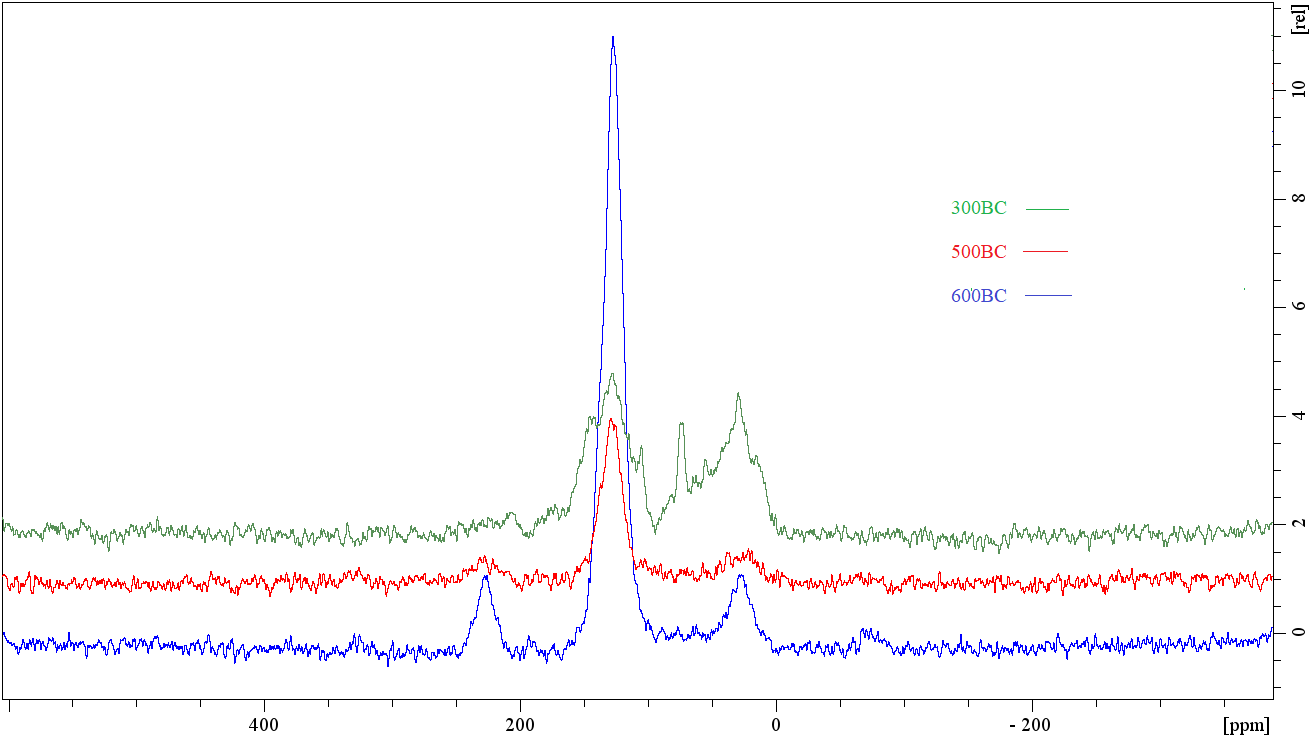


Figure S2. ^13^C NMR spectroscopy of biochar (produced at pyrolysis temperature 300 ℃, 500 ℃ and 600 ℃).
